# Glia as a key factor in cell volume regulation processes of the central nervous system

**DOI:** 10.3389/fncel.2022.967496

**Published:** 2022-08-25

**Authors:** Lenin David Ochoa-de la Paz, Rosario Gulias-Cañizo

**Affiliations:** ^1^Departamento de Bioquímica, Facultad de Medicina, Universidad Nacional Autónoma de México, Mexico; ^2^Asociación para Evitar la Ceguera en México (APEC), Unidad de Investigación APEC-UNAM, Mexico; ^3^Centro de Investigación en Ciencias de la Salud, Universidad Anáhuac México, Mexico

**Keywords:** brain edema, glia, cell volume regulation, astrocytes, Müller cells

## Abstract

Brain edema is a pathological condition with potentially fatal consequences, related to cerebral injuries such as ischemia, chronic renal failure, uremia, and diabetes, among others. Under these pathological states, the cell volume control processes are fully compromised, because brain cells are unable to regulate the movement of water, mainly regulated by osmotic gradients. The processes involved in cell volume regulation are homeostatic mechanisms that depend on the mobilization of osmolytes (ions, organic molecules, and polyols) in the necessary direction to counteract changes in osmolyte concentration in response to water movement. The expression and coordinated function of proteins related to the cell volume regulation process, such as water channels, ion channels, and other cotransport systems in the glial cells, and considering the glial cell proportion compared to neuronal cells, leads to consider the astroglial network the main regulatory unit for water homeostasis in the central nervous system (CNS). In the last decade, several studies highlighted the pivotal role of glia in the cell volume regulation process and water homeostasis in the brain, including the retina; any malfunction of this astroglial network generates a lack of the ability to regulate the osmotic changes and water movements and consequently exacerbates the pathological condition.

## The Cell Volume and The Regulation Processes

Most cells behave like perfect osmometers when the osmotic change begins, since several mechanisms that allow the regulation of cell volume is immediately activated. Extracellular osmolarity alterations induce anisosmotic changes, as in chronic renal failure, while isosmotic changes are due to an alteration in the intracellular content of solutes, such as the mobilization of ionic gradients on both sides of the membrane, under physiological (synaptic transmission) or pathophysiological (ischemic) conditions (McManus et al., 1995).

Under physiological conditions, the cell maintains its volume because of the orchestrated operation of two membrane transport systems: (1) the Na^+^-K^+^ ATPase, which maintains intracellular ion concentrations by expelling Na^+^ and exchanging it for K^+^, thus compensating for the osmotic gradient generated by the concentration of impermeable molecules; and (2) the selective permeability of the cell membrane to K^+^, which results in an output current of this cation through ion channels coupled to Cl^−^ a flow which functions as an accompanying ion to counteract the asymmetric equilibrium of impermeable organic ions (Armstrong, 2003; Lang, 2007).

**Figure 1:**
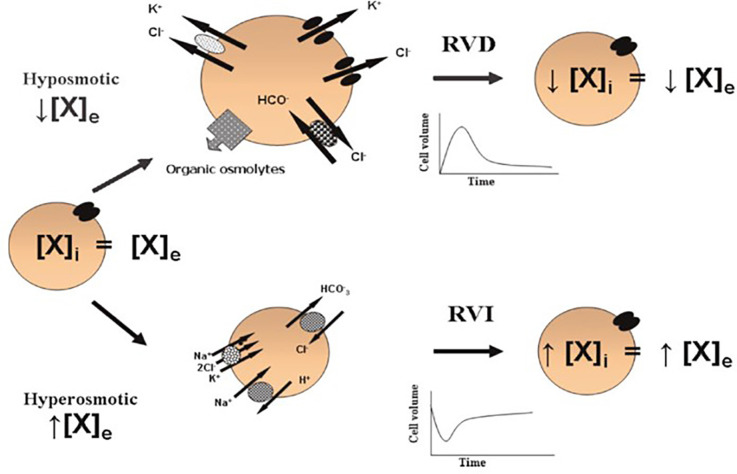
Cell volume regulation. In isosmotic conditions, the extracellular (X)_e_ and intracellular (X)_i_ concentrations of osmolytes are in equilibrium. Acute cell volume regulation is accomplished by two mechanisms: (1) regulatory volume decrease (RVD) mediated by the net loss of intracellular osmolytes in response to cell swelling induced by a hypoosmotic environment [a reduced (X)_e_ compared to the (X)_i_]; and (2) regulatory volume increase (RVI) mediated by the net accumulation of active solutes in response to cell shrinkage induced by a hyperosmotic environment [an increased (X)_e_ compared to the (X)_i_]. These phenomena allow the cells to partially recover their original dimensions and in this way, prevent the deleterious effects of changes in cell volume (taken from Franco, 2003).

Mobilization of ions (K^+^, Na^+^, and Cl^−^) and organic osmolytes (amino acids, polyalcohols) regulates cell volume. Cells respond to cell volume changes through two mechanisms: Regulatory Volume Decrease (RVD) refers to the mobilization of osmolytes towards the extracellular space in response to a volume increase, and Regulatory Volume Increase (RVI) which means an intracellular accumulation of osmolytes in response to a decrease in cell volume. Anisosmotic conditions activate both mechanisms; however, the activation of each one depends on the nature of the osmotic change (Figure 1).

In both cases, the process of regulating cell volume is divided into: (1) detection of volume changes through a volume sensor; (2) generation of signaling cascades in response to the activation of the volume sensor; and (3) activation and/or regulation of pathways responsible for the mobilization of osmolytes to compensate osmotic changes (Hoffmann and Pedersen, 2006).

During RVD, initially, there is an activation of transport systems responsible for mobilizing K^+^ and Cl^−^, amino acids, polyalcohols, and methylamines. The transport systems that participate in the mobilization of K^+^ are mainly ion channels that differ in type depending on the cell type (Calloe et al., 2007; Lotshaw, 2007). Different pharmacological and biophysical data indicate a common pathway for the mobilization of Cl^−^ and organic osmolytes (amino acids) through volume-regulated anion channels (VRAC). However, there is evidence that suggests that the routes of mobilization of Cl^−^ and organic osmolytes differ depending on the nature of the osmolyte (Franco, 2003). In the case of RVI, volume compensation is mainly due to Na^+^ mobilization through specific transport systems such as the Na^+^/H^+^ exchanger, the Na^+^/K^+^/Cl^−^ co-transporter, ion channels, and the amino acid transporters dependent on Na^+^ concentration (Franchi-Gazzola et al., 2006; Pedersen et al., 2006; Lang, 2007).

## Cell Volume and Brain Edema

Water transport is an essential function associated with different cellular processes in the central nervous system (CNS). At a cellular level, water transport is associated with cell volume regulation and, therefore, with control of extracellular space dimensions. Considering the physical imposition that involves the skull on the brain, the processes associated with RVD require complete control for the proper functioning of the CNS. The movement of water through the membranes of neural and glial cells affects, mainly, the intra- and extra-cellular concentration of ions, which impacts synaptic physiology (Kimelberg, 2004).

**Figure 2:**
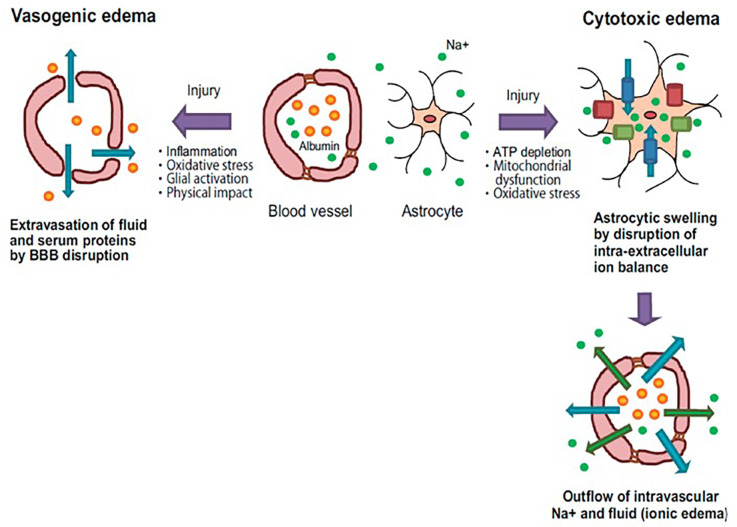
Pathology of vasogenic and cytotoxic edema. Vasogenic edema: after brain injury, endothelial tight junctions are disrupted by inflammatory reactions and oxidative stress. These events cause fluid and albumin extravasation, leading to the extracellular accumulation of fluid into the cerebral parenchyma. Cytotoxic edema: brain insults induce intracellular ATP depletion, resulting in mitochondrial dysfunction and oxidative stress. These events cause a disturbance of intra-extracellular ion balance. As a result, excessive inflows of extracellular fluid and Na^+^ into cells are induced, leading to cell swelling. Blue arrows: water flow; Green arrows: Na^+^ flow; Orange spheres: albumin; Green spheres: Na^+^; Blue columns: water channel; Green columns: ion transporter; Red columns: ion channel (taken and modified from Michinaga and Koyama, 2015).

In physiopathological conditions, the movement of water in the CNS is vital given the mortality and morbidity caused by cerebral edema. Cerebral edema is classified as vasogenic edema, where the permeability of the blood-brain barrier is compromised, generating an accumulation of extracellular water; and cytotoxic or cellular edema, due to a constant entry of water into the intracellular space (Unterberg et al., 2004; Figure 2). A large amount of experimental evidence indicates that this phenomenon represents the final point of different neurological factors where there are structural, functional, cellular, and molecular changes in the blood-brain barrier, changes in microcirculation, and alterations in the mechanisms of cell volume regulation (Vajda et al., 2002; Manley et al., 2004).

In the brain, as in other tissues, water is mobilized through plasma membranes by aquaporins, co-transport systems coupled to the mobilization of ions and organic molecules (e.g., amino acids), and a simple diffusion mechanism that has an intrinsic very slow diffusion speed. The contribution of these mechanisms depends on factors such as density, expression level, and the capacity of water flow through aquaporins and co-transporters. However, to understand the complexity of water transport in the brain, it is necessary to consider that water movement relates directly or indirectly to neuronal activity, which generates temporal changes in the extracellular space.

## Glial Cells and Cell Volume Homeostasis

In the CNS, glial cells are classified into astrocytes, oligodendrocytes, microglia, and ependymal cells. Müller cells (MC) are part of the glial component in the retina along with astrocytes and microglia. Even though all cells are perfect osmometers, glial cells and particularly astrocytes and MC play a pivotal role in the mobilization of osmolytes and water in the direction required to counteract the osmotic change. Astrocytes and MC are strategically located between the vasculature and the synaptic structure, sharing functional and neurochemical properties with neuronal cells and regulating the homeostasis of extracellular fluids through several membrane proteins responsible for the active and passive transport of ions, organic osmolytes, and osmotically obligated water (Bormann and Kettenmann, 1988; O’Neill, 1999; Simard and Nedergaard, 2004; Pasantes-Morales and Vázquez-Juárez, 2012; Reed and Blazer-Yost, 2022). In this work, we are considering the systems responsible for cell volume control points in astrocytes and MC as key players in water homeostasis in the brain and retina (Nicchia et al., 2004; Simard and Nedergaard, 2004; Bringmann et al., 2005; Kuhrt et al., 2008).

## Cell Volume Regulation, Ion Channels, and Aquaporins

### K^+^ channels

Astrocytes form a syncytium for rapid redistribution of extracellular K^+^ concentration in areas of high synaptic activity, through the high permeability they have to this cation (Sontheimer, 1992; Larsen et al., 2014). Kir 4.1 is a weak rectifying channel with the highest conductance in astrocytes, and allows bidirectional movement of K^+^ depending on the transmembrane gradient of this cation; this enables and allows astroglial K^+^ reuptake from the extracellular space, after high neuronal activity or ion accumulation by the Na^+^-K^+^ ATPase pump (Butt and Kalsi, 2006; Larsen et al., 2014). Kir 4.1 expression has been observed in the astrocytic projection that is in contact with synaptic areas, blood vessels, and the pia mater (Higashi et al., 2001). These features make these channels the main pathway to mobilize K^+^ in response to a cell volume change under pathological and physiological conditions. Kir 4.1 heterodimerizes with Kir 5.1 mouse neocortex glial cells, whereas in the hippocampus Kir 4.1 is found as a homomer (Hibino et al., 2004). The relevance of these types of interactions is not yet clear. In the stratum radiatum, the presence of two-pore K^+^ channels, TREK and TWIK subtypes were determined. These channels activate in a wide range of membrane potentials, which could contribute to the high conductance that astrocytes have towards K^+^, increasing their capacity to mobilize this cation (Zhou et al., 2009).

A major function of MC in the retina is to regulate its ionic and osmotic balance. As with astrocytes, MC buffering of K^+^ concentration occurs predominantly through Kir channels. Kir 4.1 expression has been demonstrated in the perivascular projections of MC (Kofuji et al., 2002), and Kir 2.1 has been observed “accompanying” Kir 4.1; however, the functional significance of Kir 2.1 is still unknown, even though its location would suggest participation in the control of extracellular K^+^ concentration in the end-feet of MC (Kofuji et al., 2002). Skatchkov et al. (2006) determined that two-pore K^+^ channels (TASK-1 and TASK-2) participate in the cell volume regulation of MC buffering extracellular K^+^, and maintaining the membrane potential when Kir channels are blocked or downregulated under ischemic conditions or retinal detachment (Bringmann et al., 2006; Wurm et al., 2006).

### Cl^−^ channels

Cl^−^ is the most abundant anion in animal cells and different transport systems use it as an accompanying ion to neutralize cation movements. Crépel et al. (1998) characterized an outwardly rectifying anion current in astrocytes, activated by hyposmolarity and regulated by tyrosine kinases. These Cl^−^ currents were identified in different cell types, including astrocytes, called “ICl, swell” (Nilius et al., 1994; Jentsch et al., 2002). This osmosensitive anion current is induced by VRAC, involved in the astrocyte’s RVD processes (Parkerson and Sontheimer, 2004). VRAC contributes to glutamate release from astrocytes during the spreading depression event in the cortical zone of the brain (Basarsky et al., 1999) or ischemia, exacerbating the damage by excitotoxicity (Abdullaev et al., 2006). Two independent groups determined the molecular identity of VRACs. These channels are formed by subunits of the LRRC8A protein (Leucine-Rich Repeats Containing 8a Subunit A), which are part of the LRRC8E family related to pannexins (Qiu et al., 2014; Voss et al., 2014). Reports indicate a passive flow of organic osmolytes (taurine, aspartate, glutamate, glutamine, and polyols) also generated through the hypoosmotic activation of VRACs-containing LRRC8A subunits (Strange et al., 1993; Parkerson and Sontheimer, 2003; Murphy et al., 2017; Formaggio et al., 2019).

In MC, the activation of Cl^−^ currents by γ-aminobutyric acid (GABA) have similar characteristics to those observed in neurons (Bormann and Kettenmann, 1988). Other Cl^−^ currents have been associated with voltage changes, such as the ClC-2 channel, activated by hyperpolarization (Thiemann et al., 1992). There is no protein characterization of VRAC channels in MC; however, Netti et al. (2018) showed that VRAC mediated the Ca^2+^-independent release of taurine and glutamate during the RVD processes. MIO-M1, the human MC line, showed that Cl^−^ currents modulate the membrane potential in the RVD response, accompanied by a passive flux of K^+^, indicating that the RVD response in MC depends on the magnitude of hyperosmolarity (Fernández et al., 2013).

### TRPV4 channels

Transient Receptor Potential Vanilloid type 4 (TRPV4), is a nonselective cation channel activated by heat, mechanical stimuli, and changes in cell volume; it plays an important role in physiological processes and is upregulated in a variety of pathological conditions (Vennekens et al., 2008; Kumar et al., 2018). TRPV4 is expressed in adult cortical and hippocampal astrocytes (Benfenati et al., 2007a), and despite the extensive study of TRPV4 channels and their participation in astrocytic cell volume regulation, their contribution to this event remains controversial in some cases. In an ischemic animal model with trpv4^−/−^ mice, TRPV4 channels are involved in astrocytic volume regulation only *in vitro* experiments, not *in situ* (Pivonkova et al., 2018). TRPV4 channels have a protective role during ischemia-induced edema if Ca^2+^ influx was blocked with a TRPV4 antagonist; however, the RVD processes are not affected (Butenko et al., 2012; Pivonkova et al., 2018). The role of TRPV4 in the RVD could be related to the differentiation of astrocytes and the expression level of other proteins such as aquaporin 4 (AQP4; Mola et al., 2021).

TRPV4 channels associated with K^+^ channels regulate the resting membrane potential and the voltage changes occurring during the RVD in MC (Netti et al., 2017). This TRPV4 regulation of voltage membrane and RVD depends on the threshold activated by Ca^2+^ and phospholipase A_2_ activity (Toft-Bertelsen et al., 2019). Jo et al. (2015) proposed that TRPV4 is an influx pathway for Ca^2+^ to modulate the RVD, swelling, and AQP4 expression in MC. This observation confirms an interaction between the TRPV4 channel and AQP4 in MC and astrocytes associated with persistent swelling of the CNS and retina (Thrane et al., 2011; Jo et al., 2015).

## Aquaporins

Jung et al. (1994) were the first to show the presence of water channels in the CNS. None of the other members of the aquaporin family showed such a broad expression pattern in the CNS as type 4. Six isoforms of AQP4 have been characterized, all related to cell volume regulation (Moe et al., 2008). This water channel is located in the ependymal layer of the ventricular system and astrocytes (Rash et al., 1998). In the glial projections of astrocytes, AQP4 associated with α-syntrophin allows the bidirectional mobilization of water between plasma and the CNS (Amiry-Moghaddam et al., 2004). The presence of AQP4 in glial endings that are found in the abluminal membrane of the cerebral capillaries (Nielsen et al., 1997) suggests that water transport between the systemic circulation and the CNS is modulated by the membrane permeability of the astrocytic cells (Amiry-Moghaddam et al., 2003). AQP4 participates in the astrocyte swelling causally related to neural activity, promoting the re-distribution of extracellular K^+^ and osmotically obligated water, between the plasma and the cerebrospinal fluid during periods of high neural activity (MacVicar et al., 2002; Kitaura et al., 2009). Since water flow through AQP4 is bidirectional and only occurs in response to osmotic gradients, the perivascular presence of this aquaporin could have adverse effects on pathophysiological conditions that involve water accumulation in the CNS (Amiry-Moghaddam et al., 2004). For example, AQP4-knockout mice exhibit reduced post-ischemic cerebral edema and a decrease in a glial cell volume increase in response to anisosmotic conditions (Manley et al., 2000). Astrocytes from AQP4-knockout mice and astrocytes with low expression levels of TRPV4 channels lack the Ca^2+^-dependent RVD processes, suggesting that AQP4 and TRPV4 interaction are essential to initiate the RVD in astrocytes (Benfenati et al., 2011). Downregulating AQP4 by miR-29b overexpression ameliorates damage after stroke in humans and mice with cerebral ischemia, an event associated with astrocytic swelling (Wang et al., 2015). Conversely, AQP4 deletion exacerbates water accumulation and induces higher intracranial pressures in AQP4-knockout mice (Papadopoulos et al., 2004).

In mammalian retinas, AQP4 is expressed on the MC projections facing capillaries, near the vitreoretinal border region, and on high synaptic activity areas in the retina (plexiform layers), regulating water flow and ion homeostasis from the inner retina into the vitreous body and retinal capillaries (Nagelhus et al., 1998; Kofuji and Newman, 2004). This localization of AQP4 in MC is consistent with the presence of dystrophin and a-syntrophin known to be involved in AQP4 polarization compared to cortical astrocytes (Enger et al., 2012). AQP4-knockout mice showed blood-barrier impairment in the blood vessels in contact with MC projections, but not in the areas with a stronger gliotic response and where blood vessels are covered by astrocytic projections (Nicchia et al., 2016). In MC endfeet, AQP4 colocalizes with the TRPV4 channel; AQP4 and TRPV4 interact synergically in cell volume regulation, and both regulate each other’s expression (Jo et al., 2015). Hyperglycemic conditions induce an increase in AQP4 expression in MC, and AQP4 knockdown in diabetic animals exacerbates diabetic retinopathy (Cui et al., 2012; Qin et al., 2012; Picconi et al., 2019).

## Aquaporins and Ion Channel Interactions

Considering astrocytic cells as multifunctional regulatory “units” of neuronal activity, it is not surprising to consider that both ion and water channels have a polarized and co-localized distribution to form macromolecular complexes capable of regulating specific cellular processes (Nagelhus et al., 1999; Amiry-Moghaddam et al., 2003). As mentioned before, AQP4 and TRPV4 channels regulate cell volume in astrocytes and MC; however, there are other channels besides TRPV4 that interact with AQP4. Since AQP4 is predominantly expressed in astrocytes and MC, it has been proposed as an important element associated with Kir 4.1 to facilitate the movement of water through the membrane during the process of compensation of extracellular K^+^ concentration through Kir 4.1 (Nagelhus et al., 2004). Experimental work reinforces the idea of molecular interaction between AQP4 and Kir 2.1 through the dystrophin complex. AQP4 is a water channel associated with dystrophin through α-syntrophin (Amiry-Moghaddam and Ottersen, 2003); Kir 4.1 shows co-localization with α-syntrophin in astrocytes and MC (Guadagno and Moukhles, 2004; Noël et al., 2005; Connors and Kofuji, 2006). The interaction of α-syntrophin is crucial for the formation of the dystrophin-Kir 4.1 complexes in the CNS and retina (Connors et al., 2004; Connors and Kofuji, 2006). Mice with α-syntrophin deletion show delocalization of AQP4, associated with an imbalance in the regulation of the extracellular K^+^ concentration in the CNS under hypothermia (Amiry-Moghaddam et al., 2003). It is proposed that AQP4 functions as a “transducer” at the membrane level for the detection of cell volume and during the osmotic response; however, this role is not clear yet.

Despite the physiological importance of VRAC channels and AQPs in the physiology and physiopathology of glial cells, there is very little information about the functional interaction between these two channels. Benfenati et al. (2007b) used RNAi against AQP4 in a primary culture of cortical astrocytes type 1 and observed a considerable decrease in the conductance generated by Cl^−^ through VRAC, without affecting voltage-activated K^+^ currents.

The process of cell volume regulation is a fundamental homeostatic mechanism for cellular physiology and consequently for the organism. In the case of the CNS, this phenomenon is essential due to the physical delimitation imposed by the skull; therefore, any alteration of this mechanism leads to brain damage and, in extreme cases, coma and death. This is why the study and understanding of the mechanisms involved in the regulation of cell volume in the brain are vital. In other tissues such as the retina, which is part of the CNS and lacks a physical delimitation, the regulation of water homeostasis is necessary for the correct function of the visual system. At a cellular level, astrocytes and MC, besides actively participating in synaptic physiology and metabolism, are key factors in the maintenance of water homeostasis in the brain and retina. The expression of different proteins involved in ion (Kir 4.1, 2.1, TRPV4), organic osmolytes (VRAC), and water mobilization (AQP4) in their membranes, provides an effective ability to maintain a stable cell volume under physiological and pathological conditions. On the other hand, the characterization of proteins and the understanding of the processes involved in osmotic control (sensor, signaling, and effector), will allow the development of therapies aimed at controlling or avoiding the cerebral and retinal edema that occurs in different disorders.

## Author Contributions

LO-P: conceived the manuscript, literature review, resources, and draft of the manuscript. RG-C: performed writing, literature review, and editing. All authors contributed to the article and approved the submitted version.

## Funding

This work was supported by grants PAPIIT-UNAM IN221820 and Presupuesto Interno Facultad de Medicina, UNAM.

## Conflict of Interest

The authors declare that the research was conducted in the absence of any commercial or financial relationships that could be construed as a potential conflict of interest.

## Publisher’s Note

All claims expressed in this article are solely those of the authors and do not necessarily represent those of their affiliated organizations, or those of the publisher, the editors and the reviewers. Any product that may be evaluated in this article, or claim that may be made by its manufacturer, is not guaranteed or endorsed by the publisher.
